# Supplementary material for: Bioimaging With Fluorescent Nucleic‐Acid Aptamers for the Specific Detection and Quantification of Pseudomonas aeruginosa Alone and in Heterogeneous Bacterial Populations
Source: Microbiologyopen. 2025 Dec 22;14(6):e70202. doi: 10.1002/mbo3.70202 (PMC12723068; doi:10.1002/mbo3.70202)
Supplement: Supplementary file 1 — Supporting Data 1: Tutorials. Supporting Data 2: Macros. [file MBO3-14-e70202-s001.pdf]

SUPPLEMENTAL INFORMATION

Bioimaging with fluorescent nucleic-acid aptamers for the specific detection and quantification of *Pseudomonas aeruginosa* alone and in heterogeneous bacterial populations

Chaimae Mezouarhi[1,2,3,4], Romain Vauchelles[1], Basma Abdallah[1], Janel Régine[2], Mouna Ouadghiri[3], Hassan Ait Benhassou[4], Sandrine Pelet[1], Pierre Fechter[2], Laurence Choulier[1]✉

[1]UMR7021 Laboratory of Bioimaging and Pathologies, CNRS University of Strasbourg Illkirch, France
[2]UMR7242 Laboratory of Biotechnology and Cell Signaling, CNRS University of Strasbourg Illkirch, France
[3]Medical Biotechnology Laboratory, Medical and Pharmacy School, Mohammed V University, Rabat, Morocco
[4]Prevention and Therapeutics Center, MAScIR, Mohammed VI Polytechnic University Ben Guerir, Morocco

✉Corresponding author: laurence.choulier@unistra.fr; Tel.: +33-(0)-3-68-85-41-14.

Supplementary Data 1: Tutorials

Installation instruction

1. Install ImageJ or FIJI

Way 1 : ImageJ
- Download and install ImageJ https://imagej.net/ij/download.html
- Update ImageJ to version 1.54g or higher from the menu *Help/Update imageJ …*

Way 2 : FIJI
- Download and install FIJI https://imagej.net/software/fiji/downloads
- Update ImageJ to version 1.54g or higher from the menu *Help/Update imageJ …*

2. Install macros

Way 1
- Paste macros (*.ijm files) in the imageJ *plugins* subfolder
- Restart ImageJ to complete the process.
- Macros are now available in the Plugins menu

Way 2
- Paste macros (*.ijm files) in the imageJ *plugins* subfolder
- Navigate to Plugins -> Install -> Path to File. Restart ImageJ to complete the process.
- Macros are now available in the Plugins menu

Way 3
- Aternatively, drag and drop macro in the imageJ bar to open the macro editor
- you can run macros by clicking on the *Run* button

Acquisition

- Avoid saturation
- Avoid crosstalk between DAPI and Cyto9, it cause false pseudomonas detection
- If microscope do sequential acquistion, bacterias can move between acquisition of different channels. It cause a drift who can distort classification analysis (picture below)

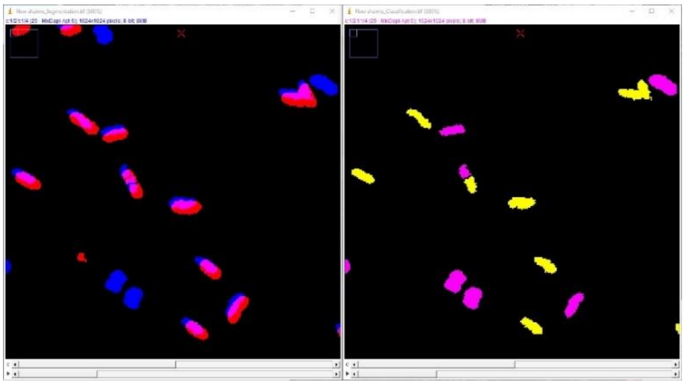

Possible solutions are :

- Use simultaneous detection of both channels
- Reduce the threshold of overlap to classify the aptamer positive cells (see in *Parameters Customisation)*

Parameters customisation

Segmentation parameters are set for 1024*1024 pixels image acquired with LSM confocal 63x. Editable settings can be modified in the script : open the macro in any text editor and change variable in the *"+++ parameters +++"* paragraph, instruction are noted as comment in the script.

```
//+++++++++++++PARAMETERS ++++++++++++++++++++++++++++++++++++++++++++++++++++++++++++++++++++++++++++++++++++++++++++++++++++++++
    TabExt =" .tsv"     ;  // results table file format : ".xls", ".tsv", ".csv" or ".txt"

    CanDapi = 1         ;  // DAPI - Staphylococcus channel #
    CanGFP  = 2         ;  // Syto9 - Pseudomonas channel #
    CanApta = 3         ;  // Cy5 - Aptamer channel #

    BactoMinSize = 25           ;  // Bacteria minimal size in pixels
    BactoMaxSize = 300          ;  // Bacteriaa maximal size in pixels
    ClassificationSeuil = 0.6   ;  // %age of overlap with aptamer signal in a segmented bacteria to be considered as postive to aptamer labelling (between 0 and 1)

    Background = 200        ;  // background level : this value is subtracted on DAPI and Syto9 image before analysis
    Seg2Gauss = 0.5         ;  // Gaussian prefilter size
    Seg2PromIndex = 500     ;  // Prominence of maxima detection for segmentation. The lower it is the more it detect objects

    TestMode = false        ;  // developper option : switch off batchmode and keep intermediate images
//++++++++++++++++++++++++++++++++++++++++++++++++++++++++++++++++++++++++++++++++++++++++++++++++++++++++++++++++++++++++++++++++++++
```

Input files

- Macros run with tif file, if images are in a microscope manufacturer format, they must be converted.

We recommand the bioformat importer tool http://www.openmicroscopy.org/bio-formats/

- Batch macro detect automatically tif files with the following extensions : *.tif , *.Tif and *.TIF

Output files

- Results are automatically saved in a new subfolder
- Measurement tabs are saved in *.tsv format
- Segmented images are saved in *.tif files
- Region of interest are saved in *.roi format and stacked in *zip files

I. Aptamers Pseudo Specificity test ROI

This first classification macro was developed to test the selectivity of aptamer for Pseudomonas bacteria.

<u>Input :</u>
3 channels image :
- channel 1 : DAPI - Staphylococcus
- channel 2 : Cyto-9 - Pseudomonas
- channel 3 : CY5 - Aptamer

<u>Process :</u>
1) All channels are segmented
2) Colocalization (surface overlap) is measured between channels 1 and and 3 between channels 2 and 3
3) Cells are classified

<u>Ouput :</u>
3 channels image for segmentation (binaries) :
- channel 1 (Blue) : DAPI - Staphylococus
- channel 2 (Green) : Cyto-9 - Pseudomonas
- channel 3 (Red) : CY5 - Aptamer

4 channels image for classified bacterias (binaries) :
- channel 1 (Blue) : Staphylococus untagged with aptamer
- channel 2 (Magenta) : Staphylococus tagged with aptamer
- channel 3 (Green) : Pseudomonas tagged with aptamer
- channel 4 (Red) : Pseudomonas untagged with aptamer

Region of interests for each staphylococcus
Magenta : Staphylococus tagged with aptamer
Yellow : Staphylococus untagged with aptamer

Region of interests for each staphylococcus
Green : Pseudomonas tagged with aptamer
Red : Pseudomonas untagged with aptamer

Result table , for each bacteria are measured :
Surface
Surface labelled with aptamer
Proportion of labelled surface
Bacteria circularity
Gobal statistics

II. Aptamers Pseudo Specificity test Batch

This classification macro was developed to test the selectivity of aptamer for Pseudomonas bacteria. It is identical to *Aptamers Pseudo Specificity test ROI* except it run analysis on all image in a folder and region of interest are not saved.

<u>Input :</u>
A folder containing 3 channels images in tif format :

- channel 1 : DAPI - Staphylococcus
- channel 2 : Cyto-9 - Pseudomonas
- channel 3 : CY5 - Aptamer

<u>Process :</u>

1) All channels are segmented
2) Colocalization (surface overlap) is measured between channels 1 and and 3 between channels 2 and 3
3) Cells are classified

<u>Ouput :</u>
Stack image with 3 binary channels from segmentation:

- channel 1 (Blue) : DAPI - Staphylococus
- channel 2 (Green) : Cyto-9 - Pseudomonas
- channel 3 (Red) : CY5 - Aptamer

Stack image with 4 binary channels of classified bacterias:

- channel 1 (Blue) : Staphylococus untagged with aptamer
- channel 1 (Magenta) : Staphylococus tagged with aptamer
- channel 2 (Green) : Pseudomonas tagged with aptamer
- channel 3 (Red) : Pseudomonas untagged with aptamer

Result table for each image (*.tsv) with classification statistics

Result table (*.tsv) with gobal classification statistics

III. Aptamers Pseudo Classifier Batch

This macro identify Pseudomonas in a bacteria culture mix, where all cells are tagged with DAPI (or any another label). It runs on all tif files of a selected folder.

Input :
A folder containing 2 channels images in tif format :
- channel 1 : DAPI - Staphylococcus
- channel 2 : CY5 - Aptamer

Process :
1) All channels are segmented
2) Colocalization (surface overlap) is measured between channels 1 and and 2
3) Cells are classified

Ouput :
Stack image with 2 binary channels from segmentation :
- channel 1 (Blue) : All bacterias
- channel 2 (Red) : Pseudomonas

Stack image with 2 binary channels of classified bacterias:
- channel 1 (Magenta) : Bacterias untagged with aptamer
- channel 2 (Yellow) : Bacterias tagged with aptamer

Result table for each image (*.tsv) with classification statistics
Result table (*.tsv) with gobal classification statistics

Supplementary Data 2: Macros

Macros developed for this study can be found at and downloaded from:

https://zenodo.org/records/15100407

This link is in open access under DOI 10.5281/zenodo.15100407
